# Efficacy of pCONUS Devices in the Management of Intracranial Aneurysms: Outcomes of 190 Patients

**DOI:** 10.7759/cureus.70075

**Published:** 2024-09-24

**Authors:** Hosam M Al-Jehani, Ahmed Hafez Mousa, May A AlHamid

**Affiliations:** 1 Department of Neurosurgery and Interventional Neuroradiology, King Fahad Hospital of the University, Dammam, SAU; 2 Department of Neurology and Neurosurgery, Montreal Neurological Institute and Hospital, McGill University, Montreal, CAN; 3 Department of Neurosurgery, Weill Cornell University, New York, USA; 4 Department of Neurosurgery and Interventional Neuroradiology, Imam Abdulrahman Bin Faisal University, Al Khobar, SAU; 5 Department of Neurosurgery and Interventional Neuroradiology, King Fahad Specialist Hospital, Dammam, SAU; 6 Department of Neurology and Interventional Radiology, King Fahad Hospital of the University, Imam Abdulrahman Bin Faisal University, Dammam, SAU

**Keywords:** intracranial aneurysms (ias), pconus device, systematic review, therapeutic guidelines, wide-necked bifurcation aneurysms

## Abstract

Intracranial aneurysms (IAs) pose a significant health concern, necessitating effective treatment modalities. The pCONUS device has emerged as a promising option for managing complex IAs, particularly wide-necked bifurcation aneurysms. Evaluating its efficacy across multiple studies is essential for establishing therapeutic guidelines. A systematic review was conducted following Preferred Reporting Items for Systematic Reviews and Meta-Analyses (PRISMA) guidelines to identify studies assessing the efficacy of pCONUS devices in treating cerebral aneurysms. PubMed, Google Scholar, and Scopus were searched for relevant articles published from January 1, 2000, to December 31, 2021. Inclusion criteria encompassed clinical trials examining pCONUS device benefits for ruptured or unruptured cerebral aneurysms. Data extraction and quality assessment were performed independently by two reviewers. Out of 390 initially identified articles, eight studies met the inclusion criteria. These studies collectively involved 190 participants with intracranial aneurysms. The sample sizes ranged from seven to 40 patients, predominantly in retrospective designs. Complete occlusion rates varied from 46.8% to 100%, with a mean diameter of treated aneurysms ranging from 2.5 mm to 8.83 mm. This systematic review suggests that pCONUS devices are feasible and effective for treating complex bifurcation cerebral aneurysms, with acceptable complication rates. Despite limitations such as retrospective study designs and limited follow-up durations, the findings support the beneficial role of pCONUS devices in managing challenging intracranial aneurysms. Larger collaborative efforts with longer follow-up durations are warranted to validate these findings and establish wider therapeutic guidelines.

## Introduction and background

Intracranial aneurysms (IAs) refer to localized dilations in the cerebral arteries predominantly affecting the circle of Willis [1]. It is common among adults and is increasingly identified due to the frequent cranial imaging. Digital subtraction angiography (DSA) is the current gold standard for IA imaging and can determine its morphological features such as size, neck diameter, and delineation [2]. IA can be categorized structurally into two types: saccular (berry) and fusiform. Saccular aneurysms are more common with a form of a sac-like pocket that arises from a cerebral wall, while fusiform aneurysms are dilations that affect a short length of a vessel where the entire vessel diameter is increased [1]. Previous evidence showed a significant association between family history, female gender, hypertension, smoking, and alcohol consumption and the greatest risk of having IA [3,4]. New research employing innovative imaging techniques such as 3-Tesla magnetic resonance angiography (MRA) indicates a potentially higher prevalence of intracranial aneurysms, with 6.6% of adults aged 40-84 years showing intradural saccular aneurysms measuring ≥2 mm. This number reaches 8.3% when including smaller (≥1 mm) aneurysms and extradural aneurysms [5]. The likelihood of aneurysmal rupture largely depends on the size, location, and morphology of the lesion as well as family history. A study by Lindgren et al. concluded that irregular or multilobular-shaped IAs are strongly associated with rupture in saccular intracranial aneurysms (sIA) of all sizes and independent of the location or patient background [6]. For fusiform intracranial aneurysms (fIA), however, the initial diameter of the aneurysm was concluded to be a significant predictor of lesion rupture [7]. The treatment plan for intracranial aneurysms includes one of two options: an open surgical clipping or endovascular therapy. Endovascular therapy choices for intracranial aneurysms are rapidly evolving and developing with time. Methods may involve coiling, stent or balloon-assisted coiling, and flow diversion devices [8].

Despite the fact that these treatments were widely accepted as safe and effective treatment options for IAs, in cases of complex intracranial aneurysms such as those that are wide-necked, fusiform, or involving bifurcations, endovascular therapy may be difficult to implement due to the importance in protecting patency of parent vessels [9]. Therefore, to improve treatment results in these challenging situations, new techniques and devices have been introduced. Among the new techniques are the Y/X stenting and waffle-cone techniques [10]. In terms of devices, pCONUS devices were developed for the treatment of complicated bifurcation IAs. pCONUS is an electrolytically detachable stent for the proximal vessel with clover-shaped petals reinforced by a net of nylon fibers in the center of the construct providing a more effective border of the parent artery [11]. Individual studies reported promising findings and technical feasibility with its utilization. Sorenson et al. assessed the efficacy of pCONUS and showed that the technical success rate was 100% [12]. However, the long-term complete occlusion rate was 60% with a re-treatment rate of 14% [13-16]. Although numerous individual studies have sought to evaluate experiences with pCONUS, it is crucial to examine aggregated findings from multiple studies to establish suitable therapeutic guidelines. This meta-analysis assesses the effectiveness of pCONUS devices in managing patients with intracranial aneurysms (IAs).

## Review

Materials and methods

Overview

A systematic review was carried out to identify any studies testing the efficacy of the pCONUS device in the management and treatment of cerebral aneurysms. Title and abstract screening, full-text review, and data extraction were handled independently by two reviewers, and any disagreements were resolved by discussion and consensus. We followed the Preferred Reporting Items for Systematic Reviews and Meta-Analyses (PRISMA) protocol [13].

Search Strategy 

PubMed, Google Scholar, and Scopus databases were used to search for and identify eligible articles. The search strategy included a combination of the following terms: pCONUS device, intracranial aneurysm, treatment, management, evaluation, and effectiveness.

Word variations and exploded Medical Subject Headings were searched for whenever feasible. Additionally, reference lists were hand-searched to identify further studies of interest. Table 1 shows the exact search strategies stratified by database. The databases were used to look for eligible articles from January 1, 2000, to December 31, 2021.

**Table 1:** Search strategies and results stratified by database

Database	Search terms	Strategy	Results
PubMed	pCONUS device, intracranial aneurysm, treatment, management, evaluation, effectiveness	("pCONus device" OR "intracranial aneurysm") AND ("treatment" OR "management" OR "evaluation" OR "effectiveness")	90
Google Scholar	Same as PubMed	Same as PubMed	154
Scopus	Same as PubMed	Same as PubMed	146

Study Selection

The inclusion of studies testing the efficacy of the pCONUS device in the management and treatment of cerebral aneurysms was evaluated for the study's inclusion criteria. The following were the criteria for inclusion in the study: (1) clinical trials investigating the benefits and drawbacks of the pCONUS device for the treatment of cerebral aneurysms and (2) studies that have examined the possible function of the pCONUS device in a patient group that has either ruptured or unruptured cerebral aneurysms. The exclusion criteria included (1) all preclinical studies that discussed the role of the pCONUS device in the management of intracranial aneurysms in animals and (2) studies that discussed the use of the pCONUS device in conjunction with another stent device (e.g., waffle-cone technique, barrel device, pulse rider, and flow diverters) for evaluating the efficacy in intracranial aneurysms. Finally, for review, all of the qualifying articles that were available in the English language were chosen. Studies published before January 1, 2000, were excluded.

Data Extraction and Quality Assessment

We extracted the following information, if available, from all included publications: publication year, sample size, study design, total number of aneurysms, total number of ruptured aneurysms, total number of unruptured aneurysms, and mean diameter of the aneurysms (in mm). The risk of bias evaluation was carried out separately on each paper by two reviewers in a blinded fashion. In case of a disagreement, the senior author's judgment would guide the final score. The Strengthening the Reporting of Observational Studies in Epidemiology (STROBE) 22-point checklist was used specifically to assess the risk of bias in the retrospective cohort studies. Table 2 reports the outcomes of the quality assessment on each of the studies included [14,15,17-22].

**Table 2:** Quality of evidence of the included retrospective cohort studies STROBE: Strengthening the Reporting of Observational Studies in Epidemiology

Study number	Author	STROBE score	Assessment of quality
1	Aguilar-Pérez et al. [14]	21	High
2	Gory et al. [15]	21	High
3	Fischer et al. [17]	22	High
4	Labeyrie et al. [18]	20	High
5	Lylyk et al. [19]	19	High
6	Saeed et al. [20]	20	High
7	Ulfert et al. [21]	18	High
8	Yeomans et al. [22]	22	High

Results

Literature Search

As seen in the PRISMA flowchart in Figure 2, a total of three databases provided a net of 390 articles, which were narrowed down to eight studies [14,15,17-22] after being subjected to screening and eligibility evaluation by two independent reviewers on an individual study basis.

**Figure 1:**
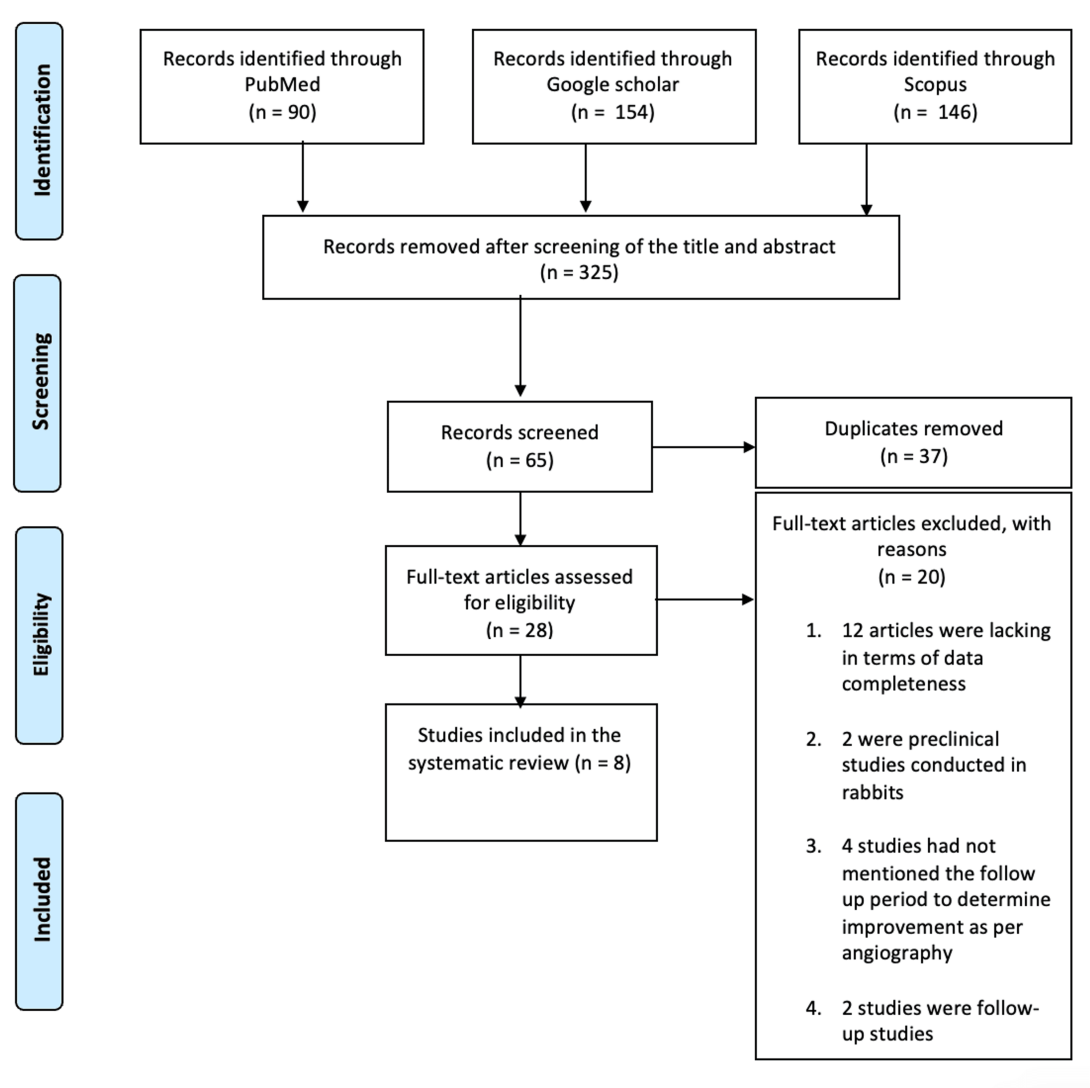
PRISMA flowchart showing the study inclusion and exclusion process PRISMA: Preferred Reporting Items for Systematic Reviews and Meta-Analyses

Study Characteristics

The use of a pCONUS device in the therapy of cerebral aneurysms was evaluated in a total of 190 research participants with intracranial aneurysms, who were involved in the eight studies that evaluated the use of pCONUS devices. The study characteristics are described in Table 3.

**Table 3:** Characteristics of the studies included

Study number	First author	Year of publication	Sample size	Study design	Total number of aneurysms	Number of ruptured aneurysms	Number of unruptured aneurysms	Mean diameter of the aneurysm (in mm)
1	Aguilar-Pérez et al. [14]	2014	28	Retrospective	28	9	19	-
2	Gory et al. [15]	2017	40	Retrospective	40	2	38	6.4 (3-11.1)
3	Fischer et al. [17]	2015	25	Retrospective	25	7	18	6 (4-13)
4	Labeyrie et al. [18]	2017	36	Retrospective	36	7	29	5.4
5	Lylyk et al. [19]	2018	12	Retrospective	12	2	10	8.83±5.83
6	Saeed et al. [20]	2017	7	Retrospective	7	4	3	8 (4.4-12.6)
7	Ulfert et al. [21]	2018	22	Prospective	22	1	21	2.5 (1.9-3)
8	Yeomans et al. [22]	2016	20	Prospective	20	4	16	-

Table 3 presents a summary of the key findings from the eight included studies. The studies varied in sample size, design, and parameters reported. The sample sizes across the studies ranged from seven to 40 patients. The majority of the studies were retrospective in nature, with only two studies employing a prospective design. The total number of aneurysms included in each study ranged from seven to 40. The number of ruptured aneurysms varied from one to nine, while the number of unruptured aneurysms ranged from 10 to 38. The mean diameter of the aneurysms was reported in five out of the eight studies. The mean diameter ranged from 2.5 mm to 8.83 mm. However, it is important to note that there was variability in the reporting format, with some studies providing a range (e.g., 4.4-12.6 mm) and others providing a mean with standard deviation (e.g., 8.83±5.83 mm). Overall, these results highlight the heterogeneity in sample characteristics and reporting standards across the included studies, which may influence the interpretation and generalizability of the findings.

Efficacy of pCONUS Devices in the Management of Intracranial Aneurysm

**Figure 2:**
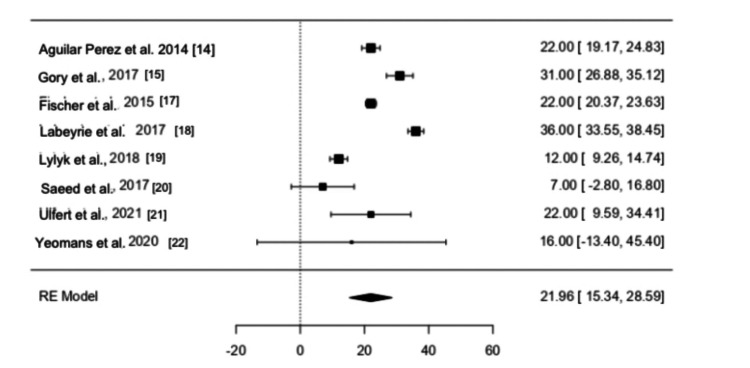
Forest plot for the utilization of pCONUS in the management of cerebral aneurysm RE: random effect

A total of 190 patients were evaluated with respect to the usage of pCONUS devices for the management of cerebral aneurysms. Out of 190 study participants across eight studies, 161 patients had complete occlusion of their wide-necked intracranial aneurysms, according to the overall summary estimate calculated from the forest plot (Figure 2). The device was found to be effective in occluding intracranial aneurysms in the majority of the patients. The use of the pCONUS device was related to the effective obliteration of broad neck cerebral aneurysms as well as relief in neurological symptoms as the total summary estimate had a p value of <0.001 and a Z score of 6.50, indicating that it was statistically significant. In addition, the 95% confidence interval for the odds ratio for the summary estimate was completely on one side of the line of zero effect, indicating the beneficial role of the pCONUS device in the management of intracranial aneurysms. The combined estimate favored the use of pCONUS devices in the management of intracranial aneurysms.

Exploration of Heterogeneity and Publication Bias

**Figure 3:**
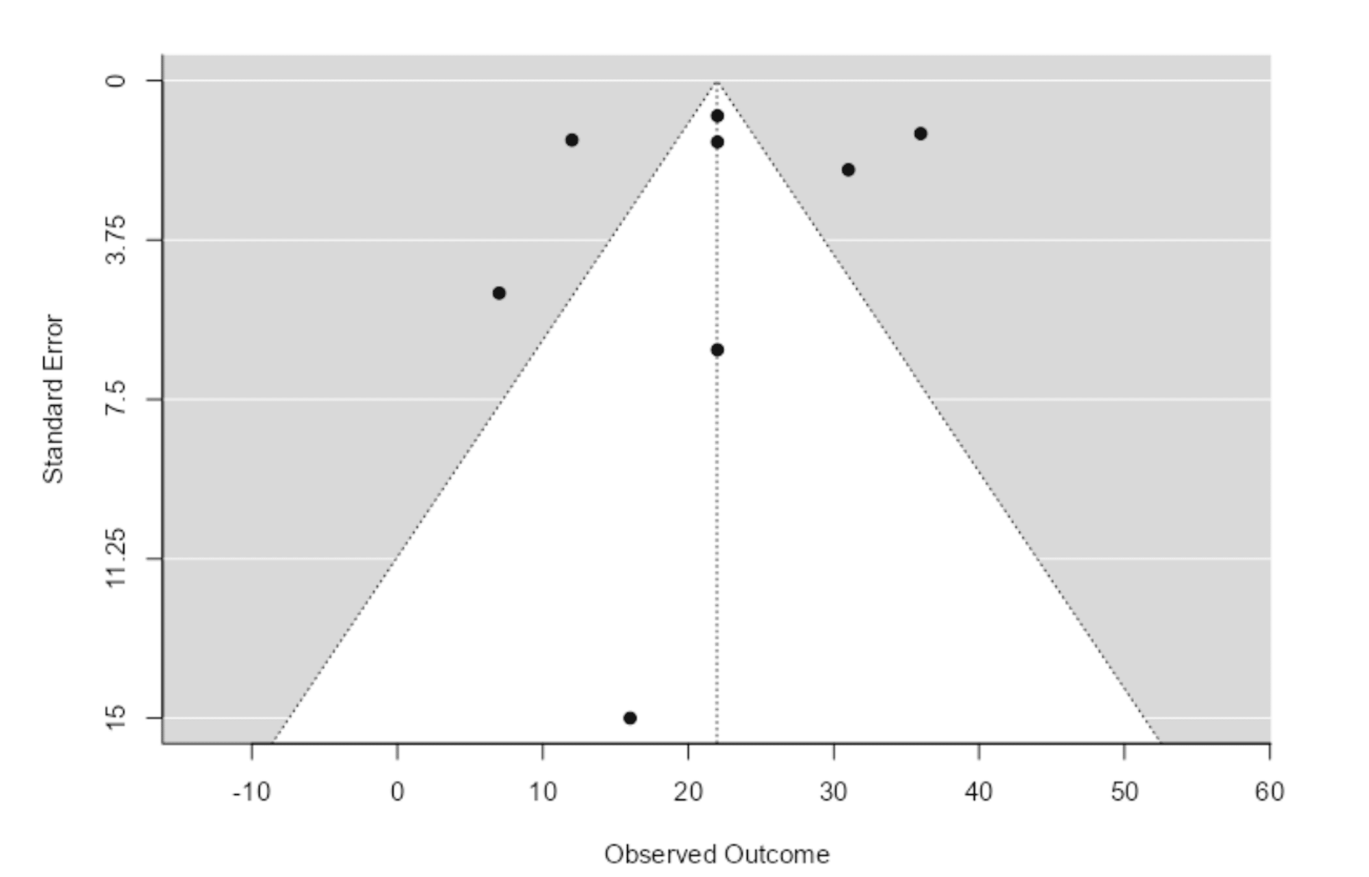
Funnel plot for publication bias and heterogeneity

In addition to the I^2^ test, Egger's test was employed to analyze the heterogeneity of the study results in this investigation. To avoid the possibility of publication bias, certain steps were taken during the research process. According to the inverted funnel plot shown in Figure 3, there is roughly equal representation of the data with respect to the utilization of pCONUS in the management of intracranial aneurysms. The I^2 ^test result of 96.33% (with a p value of <0.0001) suggested that there was significant variability across the studies that were chosen. Additionally, a large portion of the 95% confidence intervals for separate research overlapped with one another, indicating considerable study variability. The random effect (RE) model for the size of the mean was 21.96 (15.34, 28.59).

Discussion

The efficacy of the treatment of wide-necked intracranial aneurysm by pCONUS devices was assessed in the present study. A total of 190 study participants from eight retrospective studies were included. In 95 of the total cases of intracranial aneurysm, complete occlusion was reported at the time of follow-up. In 28 of the study participants, the treatment was found to be ineffective in occluding the aneurysm. Post-treatment neurological symptoms were improved significantly in the total summary reports.

These findings of the current study are comparable with the findings of a meta-analysis published by Sorenson et al. in 2019 [12]. They found that treatment with pCONUS devices was efficient in 60% of total cases of aneurysm in terms of long-term complete occlusion of aneurysm [23]. The difference in the rate of complete occlusion between the two studies seems to be due to the fact that the number of primary studies included in the present meta-analysis was eight along with 158 total participants as compared to nine in the study by Sorenson et al. with 201 participants [12].

In a more recently published meta-analysis, Krupa et al. reported that the utilization of pCONus devices as a treatment for wide-necked bifurcation intracranial aneurysm was moderately effective and safe. Immediately after the procedure, Raymond-Roy occlusion classification (RROC) I (complete obliteration of the aneurysm) was observed in 46.8%, RROC II in 32.9%, and RROC III (residual aneurysm) in 20.3% of the patients. In a short-term follow-up, the pooled prevalence estimate (PPE) of RROC I was 55%, RROC II was 29%, and RROC III was 16.1%. Poor neurological outcome was observed in 9.6% of the total of 198 patients with 200 aneurysms. Thromboembolic events were observed as the most frequent intra-procedural complications, which were observed in 12.1% of all procedures [12,16,23].

Although all the primary studies included in the current meta-analysis were retrospective studies, their findings were also comparable with prospective studies. Recently, in 2022, Aguilar-Pérez et al. conducted a pToWin study to see the effects of the treatment of wide-necked intracranial bifurcation aneurysm with the help of pCONus devices. Out of 115 patients, 75% and 65.6% showed adequate occlusion at 3-6 and 7-12 months of follow-up, respectively [11].

Wide-necked bifurcation aneurysms have been imposing a great challenge, particularly on the endovascular treatment aspects of intracranial aneurysms. Balloon remodeling, waffle-cone stenting, Y-stenting, and T-stenting are various techniques that have been developed to deal with such complicated morphology, but treating them remains challenging. pCONUS devices, pulse riders, and eCLIPs are recently developed, especially to deal with complicated aneurysms. Among all, the pCONUS is the most extensively used alternative neck bridging device. In cases of asymmetric aneurysms, the pCONUS device provides additional benefits as it has a tendency to re-center itself due to the firm connection between the distal petals and the proximal shaft. Furthermore, in aneurysms treated with these devices, the petals of the first device remain in the same position, preventing the coils from displacement [14].

With the complete occlusion rate at approximately 60% of all procedures, its usefulness has been discovered with technical limitations, and its full potential as the main treatment modality is yet to be explored. The good occlusion rates might be limited by angulation between the aneurysm and the parent vessel [21]. Researchers, in their original research and meta-analysis reports, recommended the pCONUS device as a good alternative intravascular treatment with very low procedure-related morbidity and mortality and good occlusion rates at short-term follow-ups. However, there is a lack of evidence in support of its efficacy in maintaining the long-term occlusion of aneurysm.

Limitations

The present systematic review was based on a majority of retrospective studies that are prone to reporting bias during data collection. Moreover, the average follow-up duration was relatively limited, which does not show the full picture of the long-term outcomes to be studied.

## Conclusions

Based on the reports of all included studies, the present systematic review suggests that the pCONUS treatment is feasible and effective for the treatment of bifurcated cerebral aneurysms, with acceptable complication rates. Therefore, our study supports the beneficial role of pCONUS devices in the treatment of more complicated bifurcation cerebral aneurysms. We encourage more collaborative and large-scale efforts to be done to have a wider cohort of patients with longer follow-up durations.
